# Household Catastrophic Health Expenditures in Maternal Care: A Cross‐Sectional Study From Semnan, Iran

**DOI:** 10.1002/hsr2.70426

**Published:** 2025-02-10

**Authors:** Sana Jafaei, Sayed Saeed Kassaeian, Navid Danaei, Farid Gharibi

**Affiliations:** ^1^ School of Medicine, Semnan University of Medical Sciences Semnan Iran; ^2^ Department of Community Medicine School of Medicine, Semnan University of Medical Sciences Semnan Iran; ^3^ Department of Pediatrics School of Medicine, Semnan University of Medical Sciences Semnan Iran; ^4^ Social Determinants of Health Research Center Semnan University of Medical Sciences Semnan Iran

**Keywords:** catastrophic health expenditure, maternal care, out‐of‐pocket cost, social protection

## Abstract

**Background:**

Due to its indisputable influence on maternal and child health, maternal care is among the most crucial requisites in all health systems.

**Objective:**

This study aimed to investigate the catastrophic health expenditures (CHE) in maternal care.

**Methods:**

This cross‐sectional survey included 400 pregnant women using systematic random sampling in Semnan, Iran, from July 1, 2022, to September 30, 2022. The study tool was a researcher‐made questionnaire, the content validity of which was approved by experts, and CVR and CVI values of 0.89 and 0.91, respectively. The CHE occurs when out‐of‐pocket medical expenditures account for 40% of household affordability and more, so all pregnancy‐related healthcare costs were recorded, and their ratio to the household's nonfood costs was calculated. Due to the qualitative nature of the data, a chi‐square test was performed to evaluate the statistical association between demographic and background variables with CHE.

**Results:**

The mean direct costs for maternal care was $1697, of which 48% was related to diagnostic services, 11% to various therapies, and 41% to medical treatment. The ratio of direct costs to nonfood costs was 48.67%, and 50% of pregnant women and their households suffered from CHE. Furthermore, the factors of educational status, employment status, basic health insurance, supplementary health insurance, mode of delivery, place of delivery, place of receiving care, and the woman's weight during pregnancy had statistically significant relationships with CHE (*p* < 0.05).

**Conclusions:**

The status of financial support provided to Iranian pregnant women is not desirable. The Iranian health system should reduce the incidence of CHE in maternal care by taking the following steps: (1) fully covering the costs of maternal care services by strengthening insurance facilities, (2) investing enough money in domestic procurement of diagnostic technology and medical supplements, and (3) providing high‐quality maternal care in the public sector facilities.

## 1 Introduction

The Millennium Development Goals (MDGs) helped bring attention to maternal health on a worldwide scale, leading to a 45% drop in maternal mortality between 1990 and 2013 [1]. However, this development was not equally dispersed over the globe, and many countries' metrics in this area are still below standard [1]. In low‐ and middle‐income nations, nearly all of the four million annual infant deaths and half a million annual maternal deaths are caused by complications during pregnancy [2]. In Iran, maternal mortality is 22 per 100,000 live births, neonatal mortality rate is 8.1 per 1000 live births, and infant mortality is 10.9 per 1000 live births (in 2020), demonstrating a significant disparity in indices across regions and socioeconomic ranks [3].

The evidence indicates that the availability of high‐quality, timely, and accessible healthcare can reduce maternal and newborn mortality and improve children's growth potential [4]. Women who do not obtain maternal care are eight times more likely to have a premature birth than those who do [5]. Furthermore, women who postpone maternal care until the third trimester of pregnancy have a higher risk of having a baby with low birth weight [6]. As a result, providing insufficient, delayed, and low‐quality care to pregnant women increases the risk of stillbirth, preterm birth, low birth weight, pregnancy complications, and maternal mortality [7]. Several studies have found that high healthcare costs harm patients by reducing their access to care, making it more likely that they will seek treatment from untrained providers, ultimately worsening their health [8]. Generally, maternal care is considered expensive due to the long period of care and using diagnostic services (such as laboratory, genetic, and sonography), repetitive specialist visits, medical supplements, and surgery. This imposes notable costs on mothers, especially in developing countries, because most of these services are high‐tech and require using some expensive imported devices. Thus, poor financial support can cause mothers not to receive the needed services, negatively affecting the community's health outcomes [9].

According to Iranian studies, a substantial proportion of expectant mothers (14%) do not have health insurance. Moreover, the existing health insurance plans do not cover many major costs, such as medical diagnostic tests, ultrasounds for fetal anomaly diagnosis, and cesarean delivery [10]. In Iran, only two‐thirds of expectant mothers obtain primary care, including sufficient medical visit sessions, referral to a physician, and weight and blood pressure assessment [10]. Moreover, 41% of pregnant women receive maternal care less than six times during their pregnancy, and just 27% receive care in the first trimester [11]. In addition, 45% of mothers do not have a complete vaccination, only 28.9% obtain essential care at the last medical session, 9% complete routine pregnancy tests, and 25% deliver babies through the help of a skilled person. In addition, only 5% and 7% of pregnancies receive postpartum care in the first 10 days and 10–40 days following delivery, respectively [12].

Recent research on the availability of maternal care and related indicators in different geographical regions of Iran indicated inequality in access to care facilities [13]. The results indicated that there is a significant geographical division in Iran in terms of maternal health. In contrast to those in Khuzestan, Kohgiluyeh and Boyer‐Ahmed, and Hormozgan provinces, mothers in rural areas of Tehran, Guilan, and Mazandaran are in generally good condition. In addition, the rural parts of Sistan and Baluchistan have the worst conditions in terms of maternal care and related health outcomes. These outcomes include care coverage, maternal mortality, pregnancy difficulties, infant mortality, and low birth weight [14]. Furthermore, the compliance of the availability of care facilities with health standards is substantially higher in rich geographical areas than in poverty‐stricken locations [15]. Women with adequate access to appropriate medical care experience a secure pregnancy and delivery [16], and consequently, fewer pregnancy‐related complications and maternal mortality rates are reported from these regions [13]. Besides, infants born to these mothers have acceptable health‐related outcomes in terms of birth injury, disability, low birth weight, premature birth, nutrition and breastfeeding, emaciation, developmental abnormalities, and mortality rate [15].

Given the importance of pregnant mothers' adequate access to high‐quality and timely care, it is essential to identify the financial obstacles in this regard [17]. Catastrophic health expenditures (CHE) are the indicator of financial accessibility to care, universal health coverage (UHC) status, and social protection of citizens against diseases and their complications costs [17]. A systematic review and meta‐analysis study conducted in Iran in 2020 estimated the prevalence of total CHE at 4.7%, with an increasing trend [18]. Despite the implementation of the health reform, the total CHE was estimated at 12.6, 11.8, and 29.9 in 2003, 2008, and 2015, respectively [19]. The conducted studies revealed that the CHE rate is undesirable in both acute and chronic diseases; for example, the CHE rate was estimated at 60% for hospitalized patients with COVID‐19 [17] and 54% for patients with multiple sclerosis [20]. The CHE rate in maternal care has been reported as 41%−75% in India [21, 22, 23, 24], 54% in Mali [25], 21% in Myanmar [26], and 16% in Congo [27]. However, as far as the researchers of this study investigated, there is no published study regarding the incidence rate and determinants of CHE maternal care in Iran. The current study aimed to address this gap by examining the role of CHE in maternal care.

## 2 Methods

### 2.1 Study Design and Participants

This cross‐sectional survey included 400 pregnant women in Semnan, Iran, from July 1, 2022, to September 30, 2022. The health system covers pregnant mothers in Iran during the 9 months of pregnancy. This care is provided to all mothers in the primary healthcare system (PHC), and the relevant care is provided to them by midwives and family physicians. For this reason, most mothers, especially those with good financial status, prefer to receive the care they need from gynecologists and obstetricians who practice medicine privately. Of course, the healthcare provided by the public sector is often incomplete, and mothers receiving services from them are forced to receive services such as sonography, color photography, pregnancy‐related laboratory tests, assessing genetic abnormalities, medication, and supplements from the private sector, and without coverage by basic and supplementary (private) health insurance. The participants were identified using the health information system (Sib system) of Semnan University of Medical Sciences, and all the existing 12 PHC facilities in the public sector were included. The PHC system in Iran is responsible for providing maternity care. Women who give birth in the private sector should still sign up for the Sib system so their children can benefit from the public sector's free vaccinations and other maternal and child services. So, the required information and statistics for delivered women and their children are recorded in the Sib system. Mothers were included if at least 1 month had passed since they gave birth to a healthy infant.

### 2.2 Sample Size Calculating

The formula below was used to determine the appropriate sample size for the study. The required sample size was expressed by *n*, the *Z*‐score value at the specified confidence level was denoted by *z*, the expected changes in answers were marked by *s*, and the margin of error was denoted by *d*. As a result, the sample size was determined to be 384 individuals at a 95% confidence level. Considering a forecast change of 0.5, the margin of error of 0.05, and adding 5% to the determined size (as recommended by some scientific sources), the final sample size was determined to be 400 participants.

$$n={\left(\frac{\mathrm{zs}}{d}\right)}^{2}={\left(\frac{1.96*0.5}{0.05}\right)}^{2}=\left(\frac{0.9604}{0.0025}\right)=384.16$$

### 2.3 Data Collection Tool

The study tool was a researcher‐made questionnaire with 94 questions, 50 of which collected demographic and background data, 40 questions recorded cost data, and four questions gathered income status. Initially, the costs were identified and categorized through interviews with obstetricians, gynecologists, and health administrators. Meanwhile, to identify all related sources of cost in maternal care and develop a comprehensive tool, the maternal care documents in the Sib system and similar studies were reviewed. The primary version of the questionnaire was developed using the data from similar studies and holding discussion sessions with experts to identify the demographic and background characteristics that may impact the incidence of CHE and income components. Subsequently, 10 experts evaluated and confirmed the content and face validity of the questionnaire. Using the criteria of necessity, relevance, transparency, and simplicity in a quadruple spectrum, the experts calculated the content validity ratio (CVR) and content validity index (CVI) to evaluate the content validity of each questionnaire item. In addition, the face validity of the questionnaire was determined by soliciting qualitative feedback from professionals on the drafting of questionnaire items [20]. The results acquired for the necessity criterion were used to calculate the CVR, and the mean of the results obtained for the other three criteria (according to the following formula) was used to determine the CVI [20]:

$$\text{CVR}/\text{CVI}=\frac{\text{nE}-\frac{N}{2}}{\frac{N}{2}}$$

Where nE was the number of experts who chose two positive spectrum possibilities in this calculation, and *N* was the total number of experts. Because 10 experts participated in this step, the approval score of 0.62% was the foundation for validating the questions in the questionnaire [20]. The validity was strongly confirmed by CVR and CVI scores of 0.89 and 0.91, respectively.

### 2.4 Sampling Method and Data Collection

In this study, we used systematic random sampling. At the beginning of the procedure, each eligible woman was assigned a code, and the interval between the groups was established by dividing the total number of the statistical population by the sample size. Then, a random number between 1 and 10 was selected, and the individual under examination was determined by periodically adding the group interval to that number. The participants were interviewed, and the incurred costs and household income were recorded. Each woman had a code, and selected women based on the sampling method of the study were contacted, and their referring time to the health center was determined. After completing a consent form, the women were interviewed in person and while visiting the PHC centers to receive routine care for their children (defined care for 1‐month‐old babies). Each woman was questioned individually, and her responses to all the study questions were recorded. Only four women were not referred to health centers, and the women with the next determined code were replaced and interviewed as previously contracted. Care provided by the public sector and the corresponding payments were reviewed using the Sib clinical information system to eliminate or minimize recall bias of incurred costs. We also collected the participants' demographic and background variables, incurred costs, and income status. The pregnancy period was generally 9 months, except for women who received pre‐ or post‐pregnancy health services for longer care periods.

### 2.5 Statistical Analysis

The primary variable of the study was the different types of direct medical costs related to maternal care. The costs of all pregnancy‐related phases, including pre‐pregnancy, pregnancy, childbirth, and post‐pregnancy care, were identified 1 month after birth. This period generally takes 9−11 months because some women had unintended pregnancies and did not receive pre‐pregnancy care. Additionally, some women did not receive post‐pregnancy care, either because they did not experience pregnancy complications or because they disregarded their doctor's or midwife's advice to undergo a re‐examination.

We gathered the number and kind of costs through the mothers' self‐report. Due to the lack of an all‐encompassing health record for mothers in both the public and private sectors, only the accuracy of reported costs in the public sector was examined and confirmed using the Sib system. The World Health Organization (WHO) states that CHE occurs when out‐of‐pocket medical expenditures account for 40% of household affordability and more. The specific calculation is shown in the following formula:

$$\frac{\mathrm{OOP}}{\mathrm{CTP}}\geq 0.4$$

CTP=TotalExpenditures–FoodExpenditures

OOP (out‐of‐pocket payment) refers to direct costs, and CTP (capacity to pay) indicates the household's affordability and nonfood costs [28].

The incurred direct medical costs, along with household income and expenditures, were calculated in Iranian Rials (IRR) and then changed to US dollars (USD) using the exchange rate announced by the Central Bank of Iran (1 USD = 42,000 IRR). The data were examined descriptively and analytically. The descriptive results were provided as frequency (percentage) for qualitative variables and mean (standard deviation) for quantitative variables. Due to the qualitative nature of the data, the chi‐square test was utilized to study the statistical relationship between demographic and background variables with CHE. For data adjustment, the obtained data was compared based on basic demographic and background variables using the chi‐square test. The results showed no significant difference in terms of age, educational status, occupation, benefiting basic insurance, type of basic insurance, benefiting supplementary insurance, place of care, delivery method, and delivery place (*p*'s: 0.651, 0.632, 0.256, 0.411, 0.912, 0.620, 0.148, 0.335, and 0.501, respectively) (*p* > 0.05).

### 2.6 Ethical Considerations

All participants signed an informed consent before inclusion in the study. We considered the privacy terms of all participants and guaranteed the publication of results anonymously. Also, the necessary permissions and approvals were obtained from the Semnan University of Medical Sciences (IR.SEMUMS.REC.1400.063).

## 3 Results

The mean age of participants was 30.29 (±5.59) years (age range: 18−48), and most women were housewives. Regarding education status, those with a diploma, a bachelor's degree, and those without a high school diploma held the largest shares, respectively. Three‐quarters of the participants had basic health insurance, and the majority had social security insurance; however, just 30% had supplementary health insurance. Most pregnant women received maternal care from both the public sector (affiliated with the Ministry of Health, Ministry of Welfare, Ministry of Oil and Petroleum, and military organizations) and the private sector. Half of the mothers had a vaginal delivery, and half of them had a cesarean section; however, most participants gave birth in government facilities (Table 1).

**Table 1:** Demographic and background characteristics of participating mothers.

Variables	Category	Frequency	Percentage
Age	Less than 20 years old	17	4.25
	21−30 years old	198	49.5
	31−40 years old	169	42.25
	More than 40 years old	16	4
Educational status	Illiterate	26	6.5
	Below high school diploma	109	27.25
	High school diploma	140	35
	Bachelor's degree	121	30.25
	Master's degree	2	0.5
	Doctorate	2	0.5
Occupational status	Employee	12	3
	Housekeeper	380	95
	Student	8	2
Basic insurance	Yes	295	73.75
	No	105	26.25
Type of basic insurance	Social security	368	92
	Treatment services	10	2.5
	Armed forced	17	4.25
	Other (banks, petroleum, etc.)	5	1.25
Supplementary insurance	Yes	120	30
	No	280	70
Place of care	Governmental centers	186	46.5
	Private centers	40	10
	Integration of private and public centers	174	43.5
Delivery method	Vaginal delivery	202	50.5
	Cesarean section	198	49.5
Delivery place	Public centers	367	91.75
	Private centers	33	8.25

Before pregnancy, the mean body mass index (BMI) in women was 25.13 (±4.53), with the lowest and highest BMI of 16 and 44, respectively; yet, only half of the participants had normal weight. Although around 16% of pregnant women verified the existence of underlying disorders such as diabetes and high blood pressure before the start of pregnancy, most of them reported that the conditions were under control and being treated. Only half of the mothers participated in sports before becoming pregnant. The mean weight gain of mothers throughout pregnancy was 11.59 kg (5.02), with the lowest and highest amounts being 3 and 30 kg, respectively; also, nearly two‐thirds of them experienced normal weight gain. High‐risk pregnancy occurred in approximately 14% of expectant women who became pregnant after the age of 35. About 11% of women experienced domestic violence (in social and economic forms). One‐third of pregnant women reported pregnancy problems, the most common of which were bleeding, dizziness, and urinary tract infections (Table 2).

**Table 2:** The status of mothers regarding potential risk factors of pregnancy.

Variables	Category	Frequency	Percentage
Weight before pregnancy	Underweight	16	4
	Normal	203	50.75
	Overweight	125	31.25
	Obese	56	14
Underlying disease	Yes	66	16.5
	No	334	83.5
Type of underlying disease	Cardiac diseases	5	1.25
	Hypertension	8	2
	Diabetes	16	4
	Kidney diseases	4	1
	Other diseases	39	9.75
	Co‐morbidity	4	1
Controlling the underlying disease	Yes	56	82.4
	Somewhat	12	17.6
Continuous exercise before the pregnancy	Yes	211	52.75
	No	182	45.5
	Somewhat	7	1.75
Gestational weight gain (based on the BMI^a^ before pregnancy)	Lower than the recommended range	68	17
	In the recommended range	259	64.75
	Higher than the recommended range	73	18.25
High‐risk pregnancy	Yes	56	14
	No	344	86
High‐risk pregnancy type	Older than 35 years	56	14
Domestic violence	Yes	46	11.5
	No	354	88.5
Type of domestic violence	Psychological	4	1
	Economical	29	7.25
	Social	22	5.5
Pregnancy complications	Yes	133	33.25
	No	267	66.75
Type of complications	Eclampsia	4	1
	Urinary tract infection	31	7.75
	Postpartum infection	12	3
	Gestational diabetes	10	2.5
	Dripping	9	2.25
	Bleeding	38	9.5
	Dizziness	31	7.75
	Uterine adhesions	8	2
	Re‐admission	8	2

a Body mass index.

The total direct cost was approximately $1697, of which $808 ($48%) was spent on diagnostic services, $193 ($11%) on visit fees, and $697 ($41%) on medical care. Also, no direct nonmedical costs were incurred by mothers due to the nature of care and provided social support, especially from their families. Among the diagnostic costs, the highest amounts were related to ultrasound and genetic tests, respectively. In addition, among the various care providers, the highest visit fees were paid to gynecologists, obstetricians, and pediatricians, respectively. Inpatient care and pharmacological supplements accounted for most medical care (Table 3).

**Table 3:** Direct costs incurred by mothers and their households.

Domains	Categories	Type of costs	Minimum		Maximum		Share from OOP^c^ (%)	Mean (standard deviation)	
			IRR^a^	USD^b^	IRR	USD		IRR	USD
Direct medical costs	Diagnosing services	Laboratory services	0	0	30,000,000	714.28	9.79	6,959,000 (±5,855,960)	165.69 (±139.42)
		Genetic tests	0	0	80,000,000	1904.76	17.90	12,760,000 (±14,143,280)	303.81 (±336.74)
		Sonography	0	0	55,000,000	1309.52	19.96	14,229,500 (±10,325,930)	338.80 (±245.85)
		The sum of diagnosing costs	0	0	140,000,000	3333.33	47.64	33,948,500 (±23,565,540)	808.30 (±561.08)
	Visits by care providers	GP^d^	0	0	2,000,000	47.62	0.04	32,000 (±219,580)	0.76 (±5.23)
		Obstetrician	0	0	20,000,000	476.20	7.92	5,648,100 (±4,275,120)	134.48 (±10.79)
		Midwife	0	0	1,300,000	30.95	0.01	11,300 (±103,340)	0.27 (±2.46)
		Pediatrician	0	0	21,000,000	500	3.14	2,242,500 (±4,083,430)	53.39 (±97.22)
		Infectious disease specialist	0	0	1,450,000	34.52	0.04	31,800 (±177,300)	0.76 (±4.22)
		Internist and endocrinologist	0	0	3,000,000	71.43	0.15	105,800 (±435,950)	2.52 (±10.38)
		Urologist	0	0	400,000	9.52	0.003	2000 (±28,280)	0.05 (±0.67)
		Nutritionist	0	0	1,500,000	35.71	0.01	7500 (±106,060)	0.18 (±2.52)
		The sum of visiting costs	0	0	34,500,000	821.43	11.32	8,081,000 (±6,046,650)	192.41 (±143.97)
	Treatment services	Hospitalization and surgery	0	0	150,000,000	3571.43	25.87	18,439,000 (±2,953,564)	439.02 (±70.32)
		Medication	0	0	36,000,000	857.14	0.92	660,000 (±3,796,900)	15.71 (±90.40)
		Medical supplement	0	0	45,000,000	1071.43	14.22	10,133,500 (±11,606,770)	241.27 (±276.35)
		Traditional treatments	0	0	0	0	0	0 (±0)	0 (±0)
		Home care	0	0	0	0	0	0 (±0)	0 (±0)
		Under‐the‐table payments	0	0	0	0	0	0 (±0)	0 (±0)
		The sum of treatment services	0	0	155,000,000	3690.48	41.02	29,232,500 ( ± 33,793,210)	696 (±804.60)
Direct nonmedical costs	Supportive and personal services		0	0	0	0	0	0 (±0)	0 (±0)
	Medical equipment (wheelchair, walker, etc.)		0	0	0	0	0	0 (±0)	0 (±0)
	Securing and customization of home and workplace		0	0	0	0	0	0 (±0)	0 (±0)
	Transportation to and from care centers		0	0	0	0	0	0 (±0)	0 (±0)
	Accommodation and food during treatment		0	0	0	0	0	0 (±0)	0 (±0)
	The sum of direct nonmedical costs		0	0	0	0	0	0 (±0)	0 (±0)
	Total incurred costs		0	0	201,200,000	4790.48	100	71,262,000 ± 47,538,970)	1696.71 ( ± 1131.88)

a Iranian rial.

b United States dollar.

c Out‐of‐pocket.

d General practitioner.

The average monthly income of households was $14,614, of which $4200 (almost 30%) was spent on nonfood costs. The mean ratio of direct costs to nonfood costs was 48.67% (±40.20), with minimum and maximum values of 0.05 and 2.09, respectively, and 50% of pregnant women and their households suffered CHE. Assessing the share of households with any OOP payments indicated that the households with 1001−200 USD with 43.5% OOP payment have the largest share of women; also, the households with a 0.2−0.4 rate of direct costs to nonfood costs with a 30.25% rate have the largest share too (Table 4).

**Table 4:** The status of OOP and the ratio of direct costs to nonfood costs in households.

Variables	The share of households	Frequency	Percentage
Out‐of‐pocket payments/direct medical costs	0−500 USD^a^	44	11
	501−1000 USD	74	18.5
	1001−2000 USD	174	43.5
	2001−3000 USD	58	14.5
	> 3000 USD	50	12.5
The ratio of direct medical costs to nonfood costs	0−0.199	79	19.75
	0.20−0.399	121	30.25
	0.40−0.599	100	25
	0.60−0.799	63	15.75
	> 0.8	37	9.25

a United States dollar.

The CHE was significantly associated with demographic and background factors, including education, employment, basic and supplemental health insurance, mode of delivery, place of delivery, place of receiving care, and maternal weight during pregnancy. Pregnant women with higher levels of education incurred higher costs than those with lower levels of education. Also, mothers who were students or employees incurred higher costs than homemakers; those who had basic and supplemental health insurance incurred higher costs than those without supplemental health insurance; mothers who had cesarean sections incurred higher costs compared to those giving birth naturally, and mothers who received care in private centers incurred higher costs than those hospitalized in public centers (Table 5).

**Table 5:** The statistical relationship between demographic/background characteristics of mothers and CHE rate.

Variables	Category	OOP^a^ (USD^b^)	Incidence of CHE^c^ (%)	*p* value
Age	Less than 20 years old	993.27	47.50	0.273
	21−30 years old	1572.86	45.45	
	31−40 years old	1856.12	54.11	
	More than 40 years old	2238.98	65.10	
Educational status	Illiterate	923.53	30.77	0.039
	Below high school diploma	1250.12	45.45	
	High school diploma	1625.59	44.28	
	Bachelor's degrees	2355.99	63.33	
	Master's degree	1714.28	100	
	Doctorate	1714.28	100	
Occupational status	Employee	2464.68	83.33	0.030
	Housekeeper	1672.44	47.89	
	Student	1697.61	100	
Basic insurance	Yes	1877.37	55.41	0.015
	No	1182.51	36.43	
Type of basic insurance	Social security	1066.01	53.73	0.306
	Treatment services	1843.45	60.00	
	Armed forced	2938.09	67.77	
	Other (banks, petroleum, etc.)	1793.12	60.00	
Supplementary insurance	Yes	2324.28	81.66	< 0.001
	No	1427.75	36.43	
Place of care	Public centers	1230.64	33.33	< 0.001
	Private centers	3149.78	90	
	Integration of private and public centers	1860.89	58.62	
Delivery method	Vaginal delivery	1230.64	32.68	< 0.001
	Cesarean section	2172.20	67.67	
Delivery place	Public centers	1543.24	47.54	0.020
	Private centers	3348.76	76.47	

a Out‐of‐pocket.

b United States dollar.

c Catastrophic health expenditures.

Among the potential risk factors of pregnancy, only gestational weight gain had a significant association with the incidence of CHE. Therefore, mothers with normal gestational weight gain experienced a lower rate of CHE than those with lower or higher weight gain (Table 6).

**Table 6:** The statistical relationship between potential risk factors of pregnancy of mothers and CHE rate.

Variables	Category	OOP^a^ (USD^b^>)	Incidence of CHE^c^ (%)	*p* value
Weight before pregnancy	Underweight	881.96	55.00	0.123
	Normal	1679.17	48.55	
	Overweight	1914.33	57.14	
	Obese	1503.12	60.71	
Underlying disease	Yes	1476.58	44.39	0.253
	No	1740.21	52.09	
Cardiac diseases	Yes	1234.52	52.38	0.497
	No	1701.38	49.49	
Hypertension	Yes	530.95	54.11	0.121
	No	1720.50	50.50	
Diabetes	Yes	1142.02	50.00	0.279
	No	1719.82	51.04	
Kidney diseases	Yes	1809.52	50.00	0.497
	No	1695.57	50.50	
Other diseases	Yes	1859.90	50.00	1.000
	No	1678.58	50.00	
Co‐morbidity	Yes	535.71	50.00	0.497
	No	1708.44	50.50	
Controlling the underlying disease	Yes	1722.09	42.85	1.000
	Somewhat	1618.99	44.63	
Continuous exercise before the infection	Yes	1660.03	48.57	0.099
	No	1766.52	53.84	
	Somewhat	1071.42	49.20	
Gestational weight gain	Lower than the recommended range	1718.90	48.06	0.036
	In the recommended range	1598.66	38.23	
	Higher than the recommended range	2018.17	67.56	
High‐risk pregnancy (pregnancy after 35 years of age)	Yes	2267.77	60.71	0.308
	No	1603.75	48.25	
Domestic violence	Yes	1091.40	56.52	0.658
	No	1775.39	49.15	
Psychological	Yes	1695.23	50.00	0.497
	No	1696.72	48.98	
Economical	Yes	914.79	50.00	1.000
	No	1755.56	50.00	
Social	Yes	969.69	36.36	0.597
	No	1739.02	48.48	
Pregnancy complications	Yes	1560.15	48.48	0.881
	No	1763.97	50.74	
Eclampsia	Yes	1958.57	50.00	0.497
	No	1694.06	50.50	
Urinary tract infection	Yes	2309.36	60.00	0.593
	No	1647.03	52.18	
Postpartum infection	Yes	2263.25	46.63	0.683
	No	1679.19	49.48	
Gestational diabetes	Yes	2527.61	60.00	1.000
	No	1675.40	60.00	
Dripping	Yes	1404.76	50.00	1.000
	No	1702.67	50.00	
Bleeding	Yes	1172.93	42.10	0.631
	No	1751.69	50.82	
Dizziness	Yes	680.95	46.66	0.105
	No	1779.07	51.89	
Uterine adhesions	Yes	1255.95	50.00	0.121
	No	1705.71	48.97	

a Out‐of‐pocket.

b United States dollar.

c Catastrophic health expenditures.

## 4 Discussion

The present study investigated the incidence of CHE due to maternal care and its determinant factors. The mean direct medical cost due to maternal care was $1697. The ratio of direct medical costs to nonfood household costs revealed that 50% of the women and their families suffered CHE. According to previous studies, this amount was 41% [21], 51% [22], 56% [23], and 75% in various regions of India [24], 54% in Mali [25], 21% in Myanmar [26], and 16% in Congo [27]. Therefore, the CHE rate in Iran is comparable to or worse than in countries with poor economies. It is worth noting that this study was conducted in Semnan province, which has a small population and adequate facilities; it is also the best Iranian province in terms of the misery index, which is calculated by combining the employment situation and inflation. Thus, other Iranian provinces seem to have a more severe problem in this regard.

This study examined the association between demographic and background factors with CHE incidence. The results revealed that pregnant women with higher levels of education incurred higher costs than those with lower levels of education. Also, mothers who were students or employees incurred higher costs than homemakers; women who had basic and supplemental health insurance incurred higher costs than those without supplemental health insurance; mothers who had cesarean sections incurred higher costs compared to those giving birth naturally; mothers who received care in private centers incurred higher costs than those hospitalized in public centers; and mothers with unusual weight gain incurred higher costs than those without weight gain. The predominance of CHE in educated and working people can be attributed to their postponement of marriage and the beginning of economic activity, which is a severe issue given the society's excessively inflationary economic conditions. Another reason for this assumption is that while 67% of working mothers over the age of 35 have high‐risk pregnancies, only 12% of homemakers experience such pregnancies.

Another notable finding of this study is the increased prevalence of CHE among mothers with basic and supplementary health insurance, which can be attributed to the limited and insufficient coverage of maternal care (particularly in private clinics) by these insurance types. Additional research indicated that low‐income pregnant women who paid significant premiums for basic and supplemental health insurance did not receive reimbursement for their care costs. Yet, wealthy families were shielded from comparable catastrophic medical bills despite not having insurance. Cesarean deliveries are more expensive than natural birth because of the nature of the surgery and the fact that most cesareans are performed in private hospitals, which have a higher cost and tariff. Approximately 45% of births in public hospitals and more than 88% of births in private hospitals are delivered via cesarean section. Mothers who have experienced excessive weight gain are at a higher risk of experiencing CHE due to their increased need for medical attention, which may include more expensive procedures such as ultrasounds.

Goli in India discovered that characteristics such as household income, degree of education, kind of care, and place of residence contributed to the occurrence of CHE [22]. In India, Mohanty showed that rural women, women from impoverished households, and women who gave birth in private facilities faced CHE substantially more than other mothers [23]. In India, Sharma's analysis identified private hospital treatment as the primary cause of CHE [24]. In the study conducted by Fournier in Mali, the location of pregnant women in remote rural areas and cesarean birth were identified as the factors impacting the prevalence of CHE [25]. In Moe Myint's study on CHE in Myanmar, factors such as mother's employment, household size, frequency of medical visits, and receiving care from experienced health professionals were found to have a significant effect on CHE rate [26]. In the study conducted by Ntambue in Congo, factors such as household income, mother's age, marital status, and giving birth in a public hospital with experienced staff had an impact on the incidence of CHE [27]. In the study of Demir in Turkey, the factors of household's annual income, type and size of households, age, and benefiting from supplementary health insurance were introduced as the main determinates of occurring CHE [29]. A glimpse of this study's findings reveals a remarkable similarity between the findings of these studies and those of the current investigation.

Based on the findings of this study, the researchers propose increasing the effectiveness of basic and supplementary insurance by providing complete coverage of maternal care costs (especially diagnostic care, surgery, and medical supplements) by basic and supplementary insurances. Other recommendations include the following: improving the quantity and quality of maternal care provided by public health centers to increase public trust and utilization of women; strict supervision on cesarean sections and their indications, particularly in private centers; hiring certified nutrition experts in the PHC system to manage the rate of pregnancy weight gain, also giving nutritional assistance packages to underweight mothers to improve maternity care and ensure healthy weight gain throughout pregnancy period; providing young people with enough employment and counseling services to encourage timely marriage and pregnancy; more financial support from student and employee mothers which has higher education usually; national investment in areas such as procurement of diagnostic technology and medical supplements, which are typically imported products in many developing countries. The research team also conducted similar studies in other parts of the country to identify the national pattern of CHE in maternal health. It is recommended that researchers conduct similar studies at specific time intervals to identify existing and emerging trends. Including qualitative interviews with mothers could have provided richer context around the financial challenges faced and coping strategies used. More analysis of specific health system and insurance policy factors contributing to high OOP costs could strengthen the policy relevance.

The main limitation of this study was the lack of a centralized health information system that keeps track of all prenatal care and its associated costs. Due to the lack of such a system, the researchers had to rely entirely on the claims and perceptions of mothers. This self‐reporting approach in the data collection phase can lead to recall bias. However, the main strengths of the present study include the large sample size, the scarcity of similar studies in Iran, and the comprehensiveness of the study instrument.

## 5 Conclusion

In Iran, the prevalence of CHE in maternal care is relatively high and undesirable. This index is significantly influenced by factors such as the educational and occupational status of mothers, the place of receiving care, and gestational weight gain. This study showed that CHE does not only occur in serious acute diseases (such as trauma or severe forms of COVID‐19) or chronic and debilitating diseases (such as multiple sclerosis and diabetes) but also in maternal care, in which we are not necessarily faced with a patient but with a simple caregiver, who is prone to facing CHE. Therefore, pregnant mothers need notable attention and financial protection due to the necessity of receiving services for several consecutive months and the complexity and costly care. The Iranian health system should reduce the incidence of CHE in maternal care by taking the following steps: (1) fully covering the costs of maternal care services by strengthening insurance facilities; (2) allocating sufficient budgets for domestic procurement of diagnostic technology and medical supplements; and (3) providing high‐quality maternal care in public sector facilities. Our results can help health decision‐making institutions and policymakers in Iran design and implement appropriate and effective promotional interventions in this regard.

## Author Contributions

Conceptualization: Farid Gharibi, Sana Jafaei, Sayed Saeed Kassaeian, and Navid Danaei. Study tool development: Farid Gharibi, Sayed Saeed Kassaeian, and Navid Danaei. Data collection, input, and analyses: Farid Gharibi, Sana Jafaei, Sayed Saeed Kassaeian, and Navid Danaei. First draft: Farid Gharibi and Sana Jafaei. Critical review: Sayed Saeed Kassaeian and Navid Danaei. All authors wrote and approved the manuscript. All authors have read and approved the final version of the manuscript.

## Conflicts of Interest

The authors declare no conflicts of interest.

## Transparency Statement

The lead author Farid Gharibi affirms that this manuscript is an honest, accurate, and transparent account of the study being reported; that no important aspects of the study have been omitted; and that any discrepancies from the study as planned (and, if relevant, registered) have been explained.

## Data Availability

The data that support the findings of this study are available on request from the corresponding author. The data are not publicly available due to privacy or ethical restrictions. Farid Gharibi (corresponding author), had full access to all of the data in this study, and take complete responsibility for the integrity of the data and the accuracy of the data analysis.
